# Supplementary material for: A Survey of Vaccine-Induced Measles IgG Antibody Titer to Verify Temporal Changes in Response to Measles Vaccination in Young Adults
Source: Vaccines (Basel). 2019 Sep 19;7(3):118. doi: 10.3390/vaccines7030118 (PMC6789707; doi:10.3390/vaccines7030118)
Supplement: Supplementary file 1 [file vaccines-07-00118-s001.pdf]

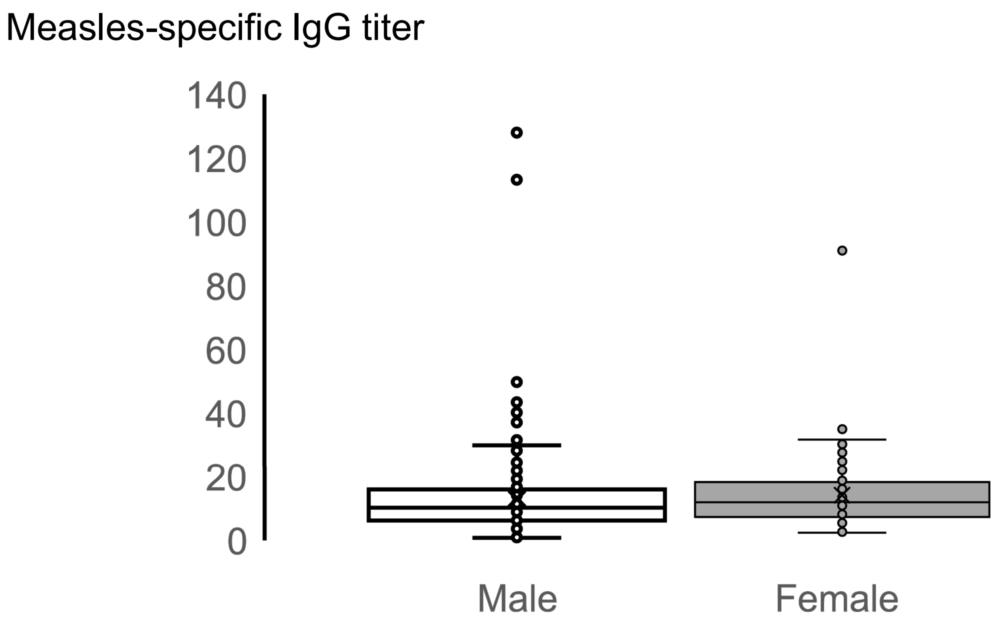


**Supplementary figure 1.** Distribution of measles-specific IgG antibody titer sorted by male and female. There were no significant differences between male and female ($P \geq 0.05$)

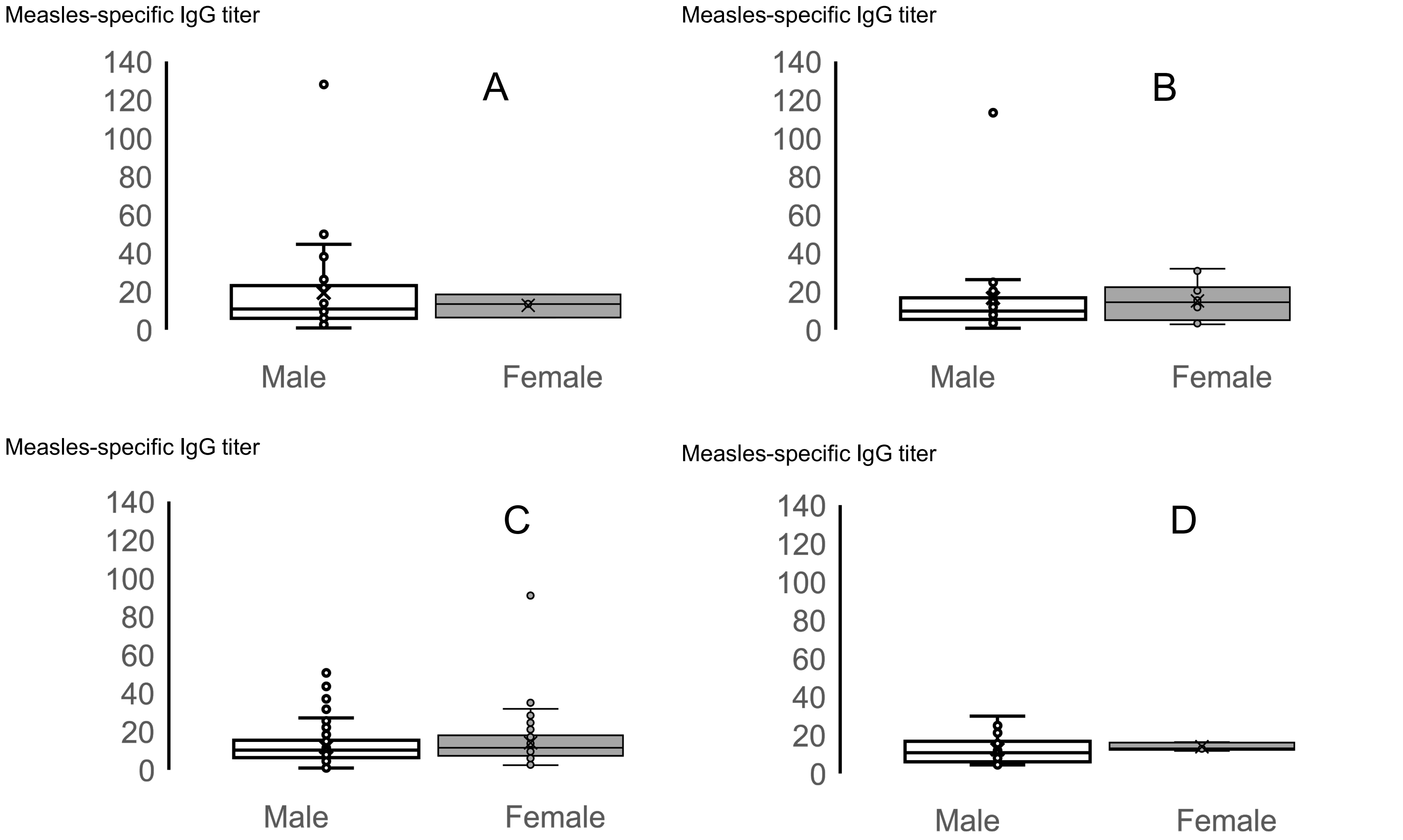


**Supplementary figure 2.** Distribution of measles-specific IgG antibody titer among the number of vaccinations sorted by male and female. A: Unvaccinated participants, B: One dose, C: Two doses, D: Three doses. There were no significant differences between male and female in all numbers of vaccinations ($P \geq 0.05$).

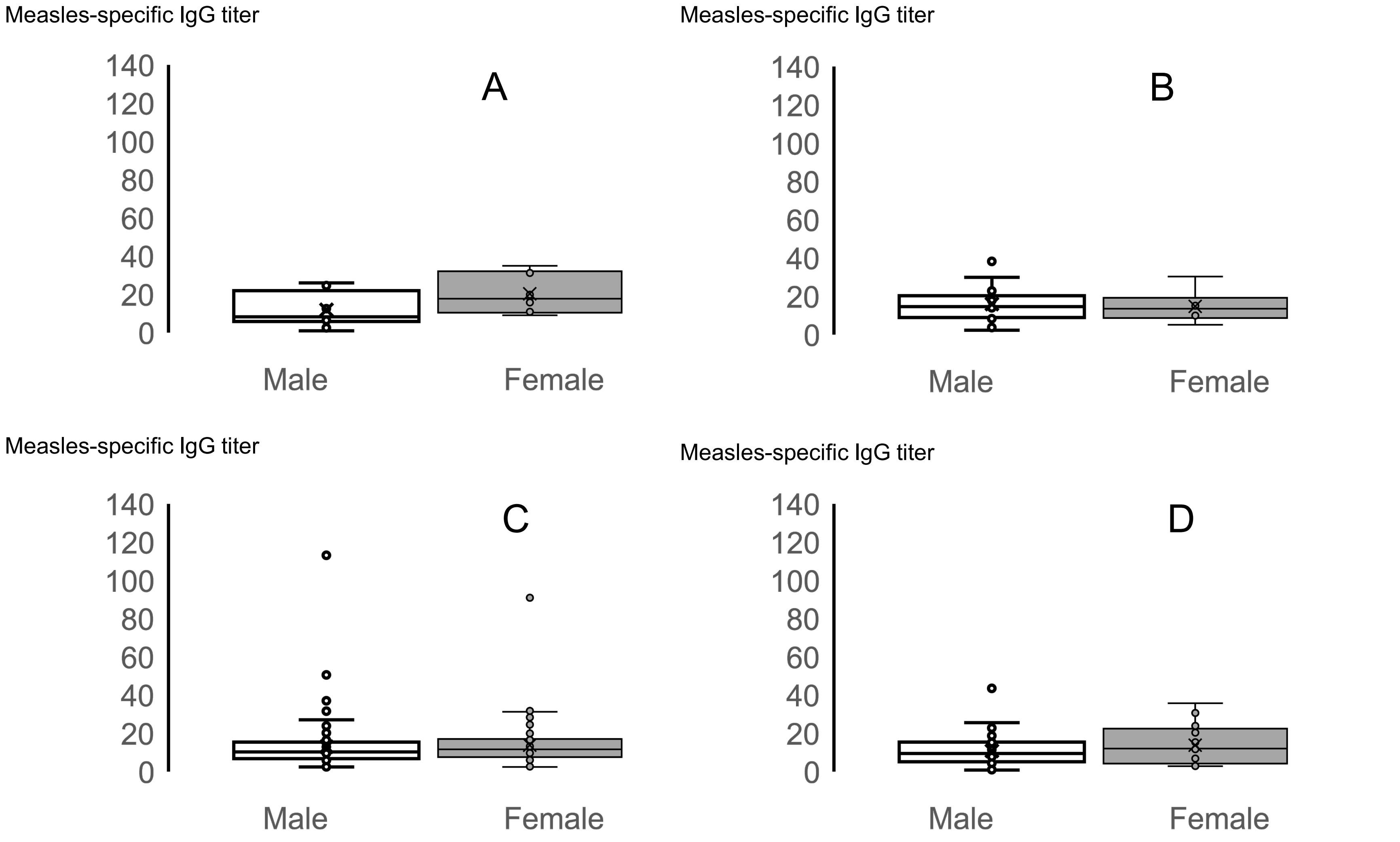


**Supplementary figure 3.** Distribution of measles-specific IgG antibody titer among four elapsed periods after last vaccinations (in months) sorted by male and female. A: ≤ 12, B: 13 - 60, C: 61 - 72, D: ≥ 73. There were no significant differences between male and female in all numbers of vaccinations ($P \geq 0.05$).
